# ADAM17 Deletion in Thymic Epithelial Cells Alters Aire Expression without Affecting T Cell Developmental Progression

**DOI:** 10.1371/journal.pone.0013528

**Published:** 2010-10-20

**Authors:** David M. Gravano, Bryce T. McLelland, Keisuke Horiuchi, Jennifer O. Manilay

**Affiliations:** 1 School of Natural Sciences, University of California at Merced, Merced, California, United States of America; 2 Department of Orthopedic Surgery and Department of Anti-aging Orthopedic Research, School of Medicine, Keio University, Tokyo, Japan; New York University, United States of America

## Abstract

**Background:**

Cellular interactions between thymocytes and thymic stromal cells are critical for normal T cell development. Thymic epithelial cells (TECs) are important stromal niche cells that provide essential growth factors, cytokines, and present self-antigens to developing thymocytes. The identification of genes that mediate cellular crosstalk in the thymus is ongoing. One candidate gene, *Adam17*, encodes a metalloprotease that functions by cleaving the ectodomain of several transmembrane proteins and regulates various developmental processes. In conventional *Adam17* knockout mice, a non-cell autonomous role for ADAM17 in adult T cell development was reported, which strongly suggested that expression of ADAM17 in TECs was required for normal T cell development. However, knockdown of *Adam17* results in multisystem developmental defects and perinatal lethality, which has made study of the role of *Adam17* in specific cell types difficult. Here, we examined T cell and thymic epithelial cell development using a conditional knockout approach.

**Methodology/Principal Findings:**

We generated an *Adam17* conditional knockout mouse in which floxed *Adam17* is deleted specifically in TECs by Cre recombinase under the control of the *Foxn1* promoter. Normal T cell lineage choice and development through the canonical αβ T cell stages was observed. Interestingly, *Adam17* deficiency in TECs resulted in reduced expression of the transcription factor Aire. However, no alterations in the patterns of TEC phenotypic marker expression and thymus morphology were noted.

**Conclusions/Significance:**

In contrast to expectation, our data clearly shows that absence of *Adam17* in TECs is dispensable for normal T cell development. Differentiation of TECs is also unaffected by loss of *Adam17* based on phenotypic markers. Surprisingly, we have uncovered a novel genetic link between *Adam17*and *Aire* expression *in vivo*. The cell type in which ADAM17 mediates its non-cell autonomous impact and the mechanisms by which it regulates intrathymic T cell development remain to be identified.

## Introduction

Proper intercellular communication is critical for the development of T cells in the thymus. Hematopoietic progenitors must receive signals from the thymic microenvironment to proliferate, differentiate, and undergo positive and negative selection [1]. Thymocyte progenitors enter the thymus at the corticomedullary junction and are identified as CD4^−^ CD8^−^ “double negative” (DN) stage. During the generation of TCRαβ^+^ T cells, they then progress through four DN stages characterized by the expression of CD44 and CD25 (DN1: CD44^+^CD25^−^, DN2: CD44^+^CD25^+^, DN3: CD44^−^CD25^+^, and DN4: CD44^−^CD25^−^) [2]. After TCRβ selection in the DN3 stage thymocytes progress through the transitory DN4 stage to the CD4^+^ CD8^+^ “double positive” (DP) stage, and then undergo both positive and negative selection prior to being released as single positive (SP) CD4 or CD8 T cells. The thymocytes migrate through anatomically distinct thymic microenvironments as they progress through these developmental stages (reviewed in [1]).

The identification of genes that mediate cellular crosstalk in the thymus during T cell development is an area of active investigation [3]. *Adam17* (Entrez GeneID: 11491; also known as TNFα-converting enzyme (TACE)), for example, has been proposed as one of these genes. The metalloprotease ADAM17 has a well characterized “sheddase” activity, in which it releases membrane bound proteins from the cell surface, often initiating their signaling ability [4], [5], [6], [7]. ADAM17 can also participate in the process of regulated intramembrane proteolysis in which it cleaves the extracellular portion of a transmembrane receptor, which leads to intracellular cleavage by the γ-secretase complex and resultant cellular signaling [8]. By analyzing the temporal and spatial activities of ADAM17, we can gain insights into the regulation of cellular signaling in the thymus.

ADAM17 is capable of cleaving a large number of immunologically relevant substrates (reviewed in [9]). It was initially described for its role in shedding the transmembrane precursor of soluble TNFα [4], [10]. Subsequently, it was described to regulate cell surface expression of TNFR-I [11], TNFR-II [12], Notch [13], Delta-like-1 [14], SCF [15], CD40 [16], L-selectin [17], CX3CL1 [18], LAG3 [19], Flt3L [20] and several other transmembrane proteins that are relevant to immune system development. Through a conventional knockout (KO) approach, it became clear that ADAM17 is critically important for the development of epithelial tissues via its regulation of the EGFR signaling pathway [17]. ADAM17 acts to shed EGF family ligands, a mechanism that is implicated in the development of mammary epithelium, heart valves, skin, and hair [17], [21], [22]. However, due to perinatal lethality of *Adam17* ^ΔZn/ΔZn^ conventional knockout mice (in which mutated ADAM17 that lacks its critical zinc binding ability is expressed), analyses of adult physiology could not be assessed. Conditional knockout of *Adam17* using the Cre/Lox system has recently solved this issue and revealed *Adam17*'s importance in inflammation [23].

The role of ADAM17 in adult T cell development is largely unknown at a mechanistic level. The current literature describes a block from the DN to DP stage of thymopoiesis in *Adam17* ^ΔZn/ΔZn^ mice that survive to adulthood on a mixed B6 and 129S3 background [24], [25]. Importantly, through transplantation of *Adam17* ^ΔZn/ΔZn^ bone marrow into RAG^−/−^ recipient mice, this block was shown to be non-cell autonomous to the hematopoietic compartment [25]. This line of experimentation indicated that the likely location of ADAM17's role in T cell development as the thymic stroma. Through *in vitro* assays analyzing the effect of the cleavage of the Notch ligand Dll1, our laboratory determined that modulation of the Notch signaling pathway is not likely the causative mechanism of the defect in *Adam17* ^ΔZn/ΔZn^ mice [26].

Thymic epithelial cells (TECs) are thought to be a principal component of the thymic stroma that provides signals to developing thymocytes, and we therefore hypothesized that ADAM17 in TECs plays a key non-cell autonomous role on development of T cells. In order to assess the role of ADAM17 specifically in adult TECs, we devised a conditional knockout strategy in which *Adam17* “floxed” mice [23] were crossed with *Foxn1-Cre* mice [27]. In these ADAM17 conditional knockout mice (*Adam17/Foxn1*), *Adam17* was effectively deleted from TECs. Surprisingly, T cell development was unaffected in *Adam17/Foxn1* mice. Interestingly, analysis of TECs revealed an unaltered TEC population structure, but a strong reduction of *Aire* mRNA expression. The lack of T cell phenotype in these mice requires a re-assessment of the proposed role of ADAM17 in the thymus.

## Results

### Generation of TEC-specific Adam17 knockout mice

**Figure 1:**
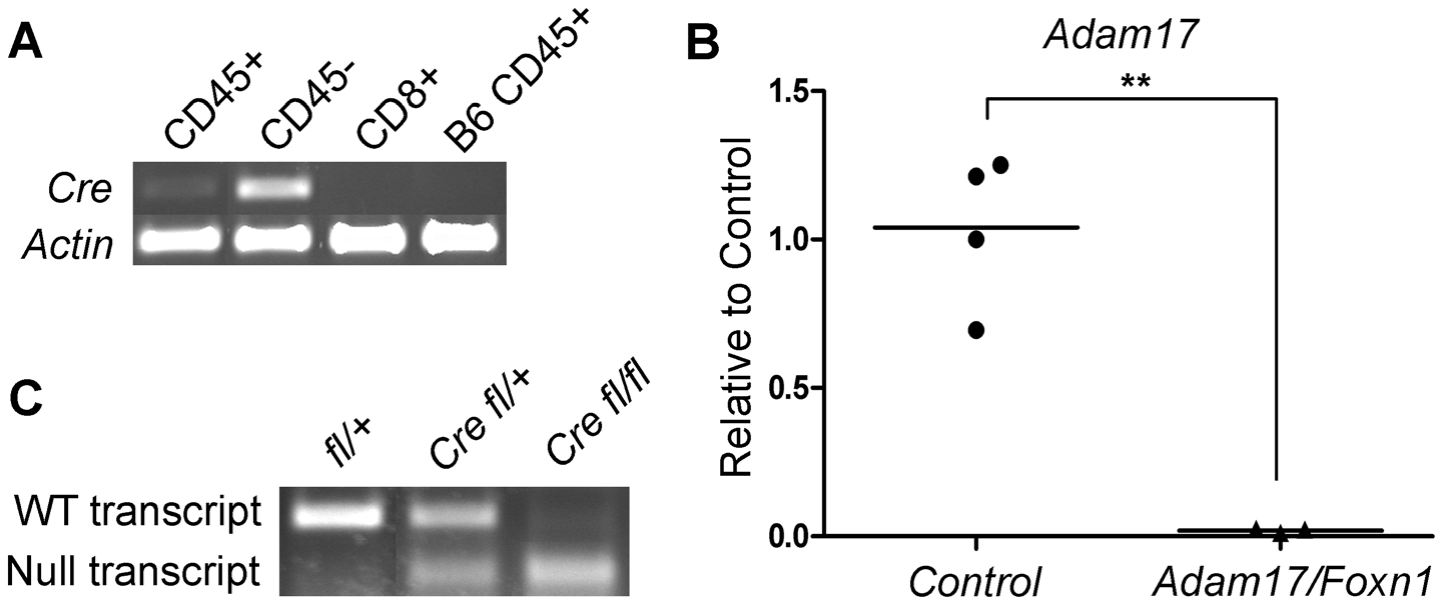
ADAM17 is efficiently deleted in TECs from Adam17/Foxn1 mice. (A) MACS-sorted CD45^+^ and CD45^-^ fractions, and FACS-sorted CD8^+^ cells of 13 pooled thymi from 1-week old *Foxn1-Cre* mice were assessed for the expression of *Cre* recombinase using *β-actin* as a loading control. MACS-sorted CD45^+^ cells from B6 thymus were used as a negative control. (B) CD45^-^Ter119^-^EpCAM^+^ TECs from 8-week old *Control* (*fl/+ or fl/fl*) or *Adam17/Foxn1* (*Cre fl/fl*) mice were assessed for *Adam17* levels by quantitative RT-PCR. Each data point represents a pool of at least 3 thymi of the indicated genotype from 3 independent experiments. *Adam17* expression levels were determined relative to control mice, using *Gapdh* expression as the internal control The mean *Adam17* expression is shown (**p<0.01, Student's t-test). (C) RT-PCR analysis for the exon2-deleted null transcript (180bp amplicon) or the wildtype transcript (313bp amplicon) in CD45^-^Ter119^-^EpCAM^+^ FACS sorted TECs from 8-week old mice. Mice were pooled to obtain ample genetic material for RT-PCR: *fl/+*, n = 6; *Cre fl/+*, n = 4; *Cre fl/fl*, n = 6.

Mice expressing Cre under the control of the *Foxn1* promoter [27] were crossed with *Adam17* exon2-floxed mice [23]. Specificity of Cre expression in TECs has previously been demonstrated and its utility in *in vivo* functional assays has been validated [28]. To further confirm Cre restriction to the TEC compartment, we performed TEC digestions from 1 week old *Foxn1-Cre* mice. CD45^−^ and CD45^+^ fractions were separated by MACS and the CD8^+^ fraction of thymocytes was further purified by FACS. RT-PCR for *Cre* expression revealed high levels of *Cre* in the CD45^−^ fraction enriched for TECs, but low to no expression in other sorted populations (Figure 1A). Mice of the genotype *Foxn1-Cre/Adam17^flox/flox^* (referred to as *Adam17/Foxn1*) were present in expected Mendelian ratios compared to littermate controls and had no overt phenotypes or distinctive external features (data not shown), unlike *Adam17* ^ΔZn/ΔZn^ mice [17].

To confirm knockdown of gene expression, *Adam17* levels were assessed by qRT-PCR. TECs were digested, MACS enriched for CD45^−^ cells, and further sorted by FACS based on EpCAM expression. Quantitative *Adam17* levels were determined using primers specific to the floxed region of *Adam17*. EpCAM^+^ TECs from *Adam17/Foxn1* mice displayed less than 2% the *Adam17* levels of littermate controls (Figure 1B). Furthermore, RT-PCR confirmed the presence of the truncated, null form of the mRNA transcript expected after recombination in *Adam17/Foxn1* mice, while the wildtype form of the transcript was present in both *Adam17^flox/+^* and *Foxn1-Cre/Adam17^flox/+^* mice (Figure 1C).

### Thymocyte development is normal in Adam17/Foxn1 mice

**Figure 2:**
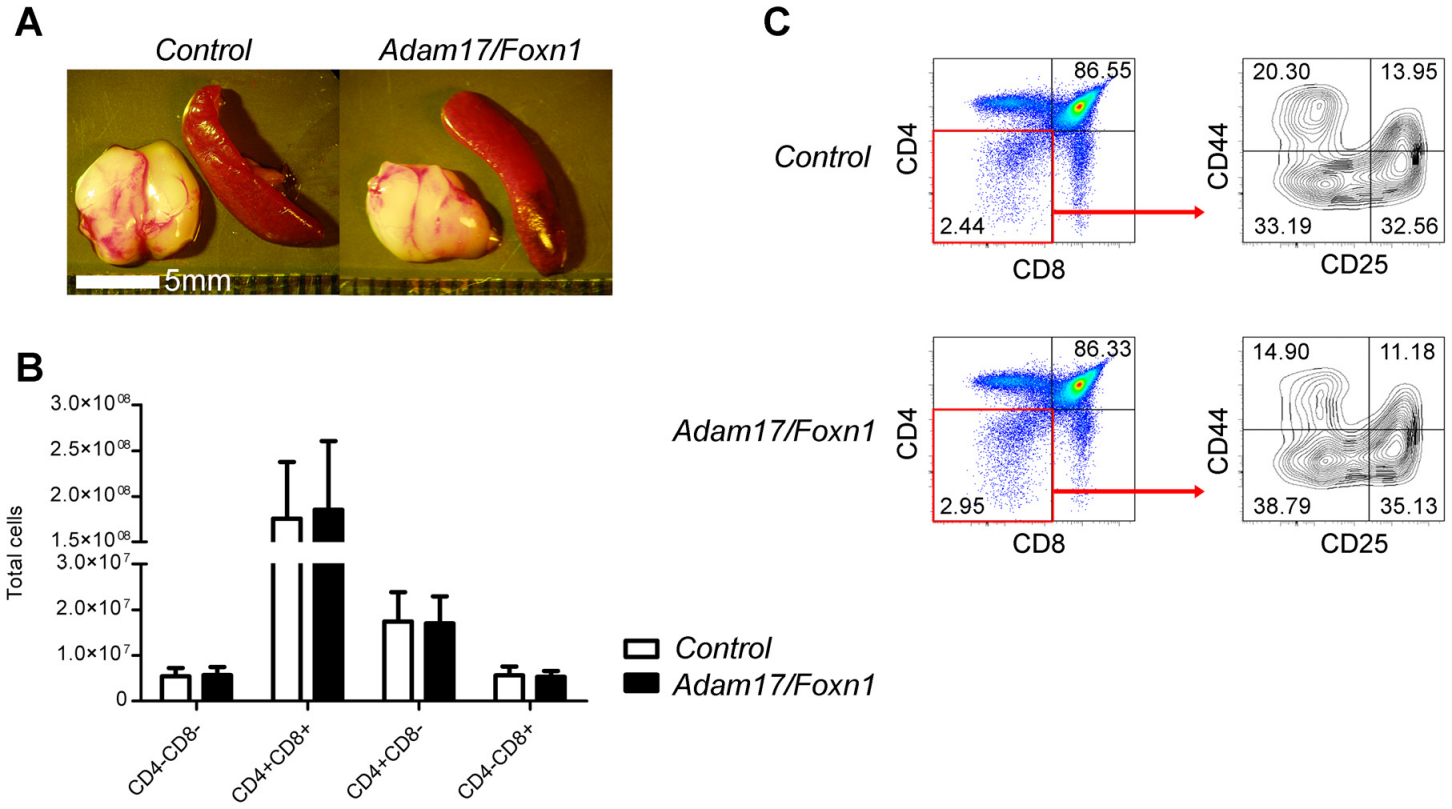
T cell developmental progression and thymocyte cellularity are unaltered in Adam17/Foxn1 mice. (A) Representative image of the thymus and spleen extracted from 4-week old *Control* or *Adam17/Foxn1* mice. (B) Total cell numbers from individual mice of the indicated genotype. Data represent mean + SD; *Control*, n = 6; *Adam17/Foxn1*, n = 8. p>0.05 for all comparisons between *Control* and *Adam17/Foxn1* thymocyte populations. (C) Representative flow cytometry data from *Control* and *Adam17/Foxn1* mice showing CD4 versus CD8 profiles and DN1 through DN4 stages, as assessed by CD44 and CD25 staining within the DN gate. *Control: fl/+ or fl/fl*; *Adam17/Foxn1: Cre fl/fl*.

Conventional *Adam17* KO mice exhibit a non-cell autonomous block in T cell development from the DN to DP stage, which indicated a likely role for ADAM17 on TECs [25]. However, in our TEC-specific *Adam17/Foxn1* knockout mice, the thymus and spleen are of normal size (Figure 2A). Likewise, analysis of intrathymic populations revealed indistinguishable numbers of DN, DP, CD4SP, and CD8SP thymocytes between control and *Adam17/Foxn1* mice (Figure 2B). Furthermore, the frequencies and absolute numbers of DN1 through DN4 stages of thymocyte development were unaltered in the absence of ADAM17 on TECs (Figure 2C, and data not shown). Likewise, analysis of regulatory T cells showed no alterations in the frequency of CD4SP thymocytes expressing FoxP3 or CD25 nor CD4SP splenocytes expressing FoxP3 (Figure S1). These results are not consistent with the hypothesis that the ADAM17 non-cell autonomous role in T cell development is localized to the TECs.

### Aire expression is diminished in Adam17/Foxn1 TECs

**Figure 3:**
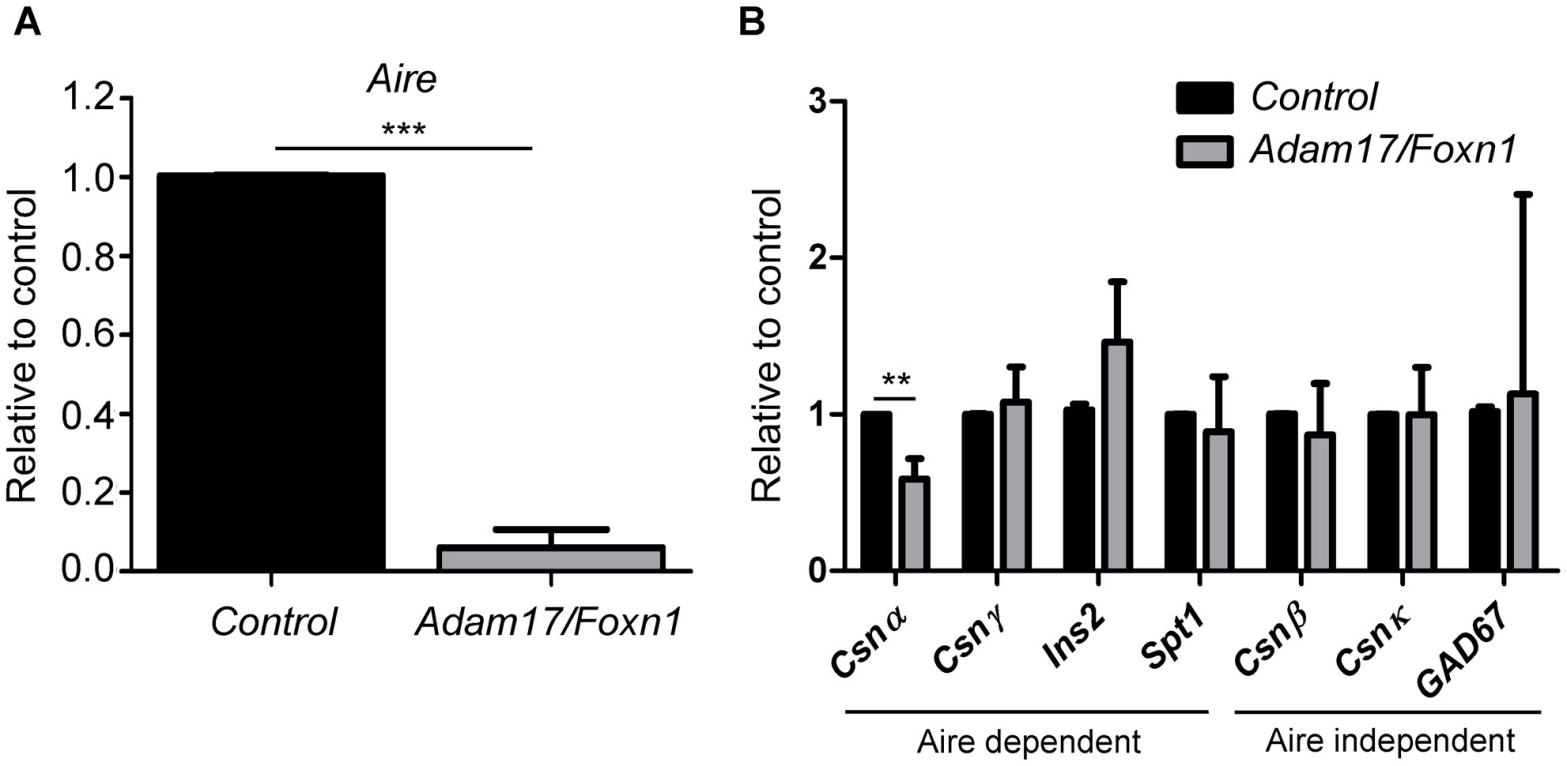
Aire mRNA expression is reduced in Adam17/Foxn1 EpCAM^+^ TECs. FACS-sorted CD45^-^Ter119^-^EpCAM^+^ TECs were pooled from 8-week old *Control* and *Adam17/Foxn1* mice (n≥3 for each pool). qRT-PCR for Aire (A) and TRA (B) expression was determined from 3 independent experiments. *Rpl7* was used as the internal control and expression levels were determined relative to mean expression in *Control* mice. Data represent mean + SD; p<0.05 (*), p<0.01 (**), p<0.001 (***). *Control: fl/+ or fl/fl*; *Adam17/Foxn1: Cre fl/fl*.

We next examined whether deletion of ADAM17 in TECs affected their development and expression of TEC-specific genes in a cell-autonomous fashion. Analysis of gene expression in FACS-sorted EPCAM^+^ TECs by qRT-PCR yielded the unexpected finding that the autoimmune regulator transcription factor, *Aire,* was diminished in *Adam17/Foxn1* mice (Figure 3A). Aire mutations have been implicated in the human disease APECED, which may result from defective medullary TEC development, and/or lack of expression of tissue-specific antigens (TRAs) that result in alterations in negative selection of thymocytes (reviewed in [29]). *Aire* mRNA expression in TECs in *Adam17/Foxn1* mice was less than 10% of control mice. TRAs whose expression is dependent or independent of Aire have been previously described [30]. The Aire-dependent TRA, *Casein α (Csnα)*, was significantly reduced in *Adam17/Foxn1* mice relative to controls (Figure 3B). However, other Aire-dependent TRAs, *Casein γ (Csnγ)*, *Salivary protein 1 (Spt1)*, *and Insulin 2 (Ins2)*, were not reduced. As expected, Aire-independent TRAs, *Casein β (Csnβ)*, *Casein κ (Csnκ)*, and *Glutamic acid decarboxylase-67 (Gad67)*, were expressed similarly between *Adam17/Foxn1* and control mice (Figure 3B).

**Figure 4:**
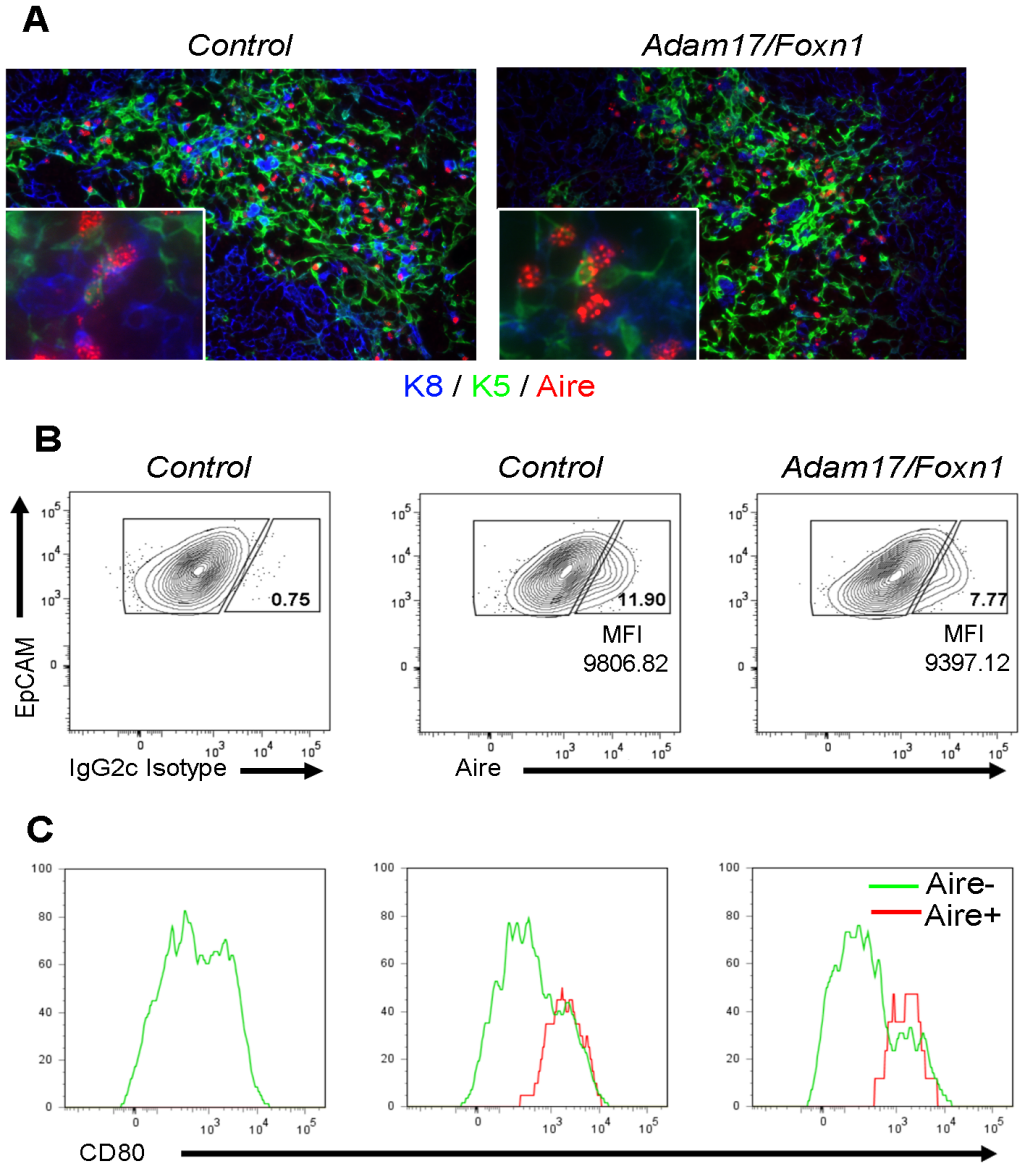
Adam17/Foxn1 mice display normal cortico-medullary thymic architecture. (A) Representative thymic sections from 8-week old mice stained for Keratin-5 (K8), Keratin-8 (K8), and Aire. Inset displays a higher magnification image demonstrating the typical speckled nuclear Aire staining [59] that is present in both *Control* and *Adam17/Foxn1* thymi. (B) Thymi from 11-week old mice were pooled and digested and CD45^-^Ter119^-^EpCAM^+^ TECs were analyzed for Aire expression (n≥3 for each pool). Aire^+^ gates were determined on the basis of IgG2c isotype control staining. (C) CD80 levels were determined for Aire^+^ and Aire^-^ TECs based on gating in Part B. MFI, mean fluorescence intensity. *Control: fl/+ or fl/fl*; *Adam17/Foxn1: Cre fl/fl*.

To assess why there was not a more pronounced reduction in Aire-dependent TRAs in *Adam17/Foxn1* thymi, we analyzed Aire protein levels by immunohistochemistry and flow cytometry. Aire protein was clearly present in *Adam17/Foxn1* thymi based on immunofluorescence analysis of thymic tissue sections (Figure 4A). Furthermore, thymi were properly organized into outer cortical and inner medullary regions as assessed by staining with the predominantly medullary marker Keratin-5 and the predominantly cortical marker Keratin-8 (Figure 4A) [31]. Morphometrical analyses of the thymic sections did not did not reveal any quantitative differences in the size of the medullary regions or the numbers of Aire^+^ cells in the medulla between controls and *Adam17/Foxn1* thymi (data not shown). Flow cytometric analysis of TECs, to measure the percentage of Aire^+^ events within the EpCAM^+^ fraction, indicated a reduction in the frequency of TECs that express Aire in *Adam17/Foxn1* thymi (Figure 4B). From two independent experiments, the frequency of Aire^+^ TECs in *Adam17/Foxn1* thymi was 65% and 85% of control mice (Figure 4B and data not shown). Furthermore, the mean fluorescence intensity of the events in the EpCAM^+^Aire^+^ gate was reduced to 92% and 96% of control levels in two independent experiments (Figure 4B and data not shown). The more modest reduction in Aire protein levels relative to the more pronounced reduction in mRNA levels may explain why Aire-dependent TRAs are not more significantly altered in *Adam17/Foxn1* thymi. To confirm the identity of Aire^+^ TECs, we stained for CD80 and observed that all Aire^+^ cells in both control and *Adam17/Foxn1* mice expressed CD80 (Figure 4C), consistent with the previous literature [32].

### Thymic stromal populations are unaltered in Adam17/Foxn1 mice

**Figure 5:**
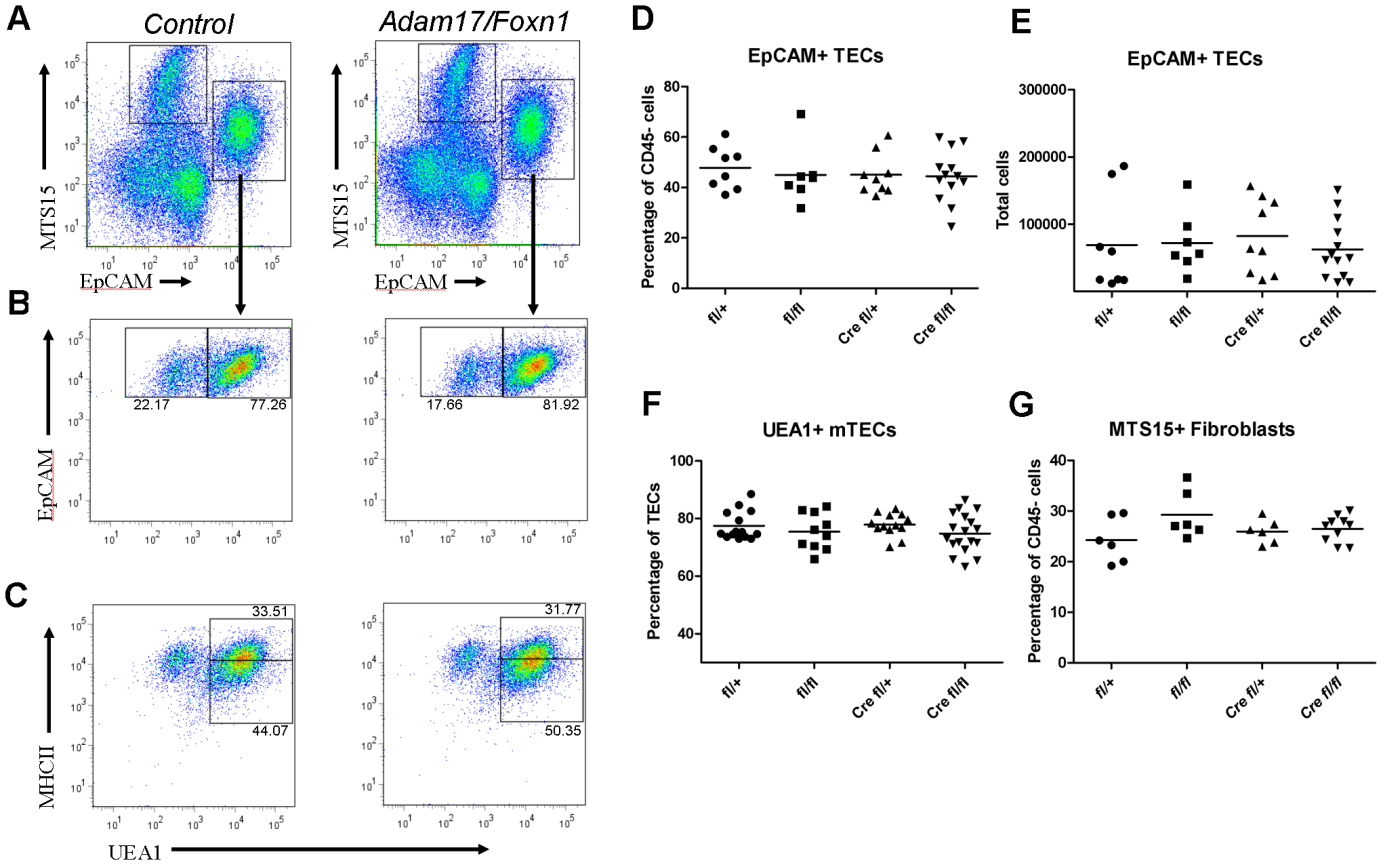
TEC population structure is intact in Adam17/Foxn1 mice. (A) Representative staining of gated CD45^-^Ter119^-^ thymic stromal cell populations from 8-week old *Control* and *Adam17/Foxn1* mice (B and C) Within the EpCAM^+^ TEC population, staining for UEA1 alone (B), and MHCII plus UEA1 (C) are shown, with frequencies of UEA1^-^, UEA1^+^, UEA1^+^MHCII^hi^, and UEA1^+^MHCII^int^ indicated. Data are representative of *Control*, n = 6 and *Adam17/Foxn1*, n = 10. (D) TEC frequencies and (E) total numbers determined based on population frequencies and total cell counts as determined by FACS. (F) Frequency of the UEA1^+^ fraction within the CD45^-^Ter119^-^EpCAM^+^ fraction. (G) Frequencies of MTS15^+^ fibroblasts. In Parts D through G, the graphs report the mean value and each data point represents an individual mouse. Data between genotypes were not statistically significant, with p>0.05. *Control: fl/+ or fl/fl*; *Adam17/Foxn1: Cre fl/fl*.

In order to determine if the observed reduction in Aire levels were the result of a reduction in mature medullary TECs (mTECs) or an alteration in another thymic population, we stained for markers of thymic stromal cells. Critically, EpCAM^+^ TECs were unaltered both in number and frequency in *Adam17/Foxn1* mice (Figure 5A, D, E). mTECs at the terminal stage of differentiation upregulate Aire expression and can be identified by several surface markers [33]. To address whether lack of ADAM17 results in a loss of mature Aire^+^ mTECs, we stained with the mTEC markers UEA1, CD80, and MHCII [34]. TEC subpopulation analysis revealed similar percentages of medullary TECs, as shown by the marker UEA1 (Figure 5B, F), and normal proportions of mature and immature medullary TEC subsets as assessed by high and intermediate MHCII levels, respectively (Figure 5C). Also, Aire^+^ TECs in both control and *Adam17/Foxn1* thymi also highly expressed CD80, corresponding to a mature medullary TEC phenotype, while Aire^−^ TECs were predominantly CD80^low^ (Figure 4C). These combined data indicate that, based on phenotypic TEC markers and histological examination, the medullary compartment in *Adam17/Foxn1* mice is normal despite the clear reduction in Aire mRNA expression. Furthermore, staining with the antibody MTS15 [35] revealed normal proportions of MTS15^+^ thymic fibroblasts in *Adam17/Foxn1* mice (Figure 5A,G).

### ADAM17 and EGF substrates are expressed on non-TEC thymic stroma

**Figure 6:**
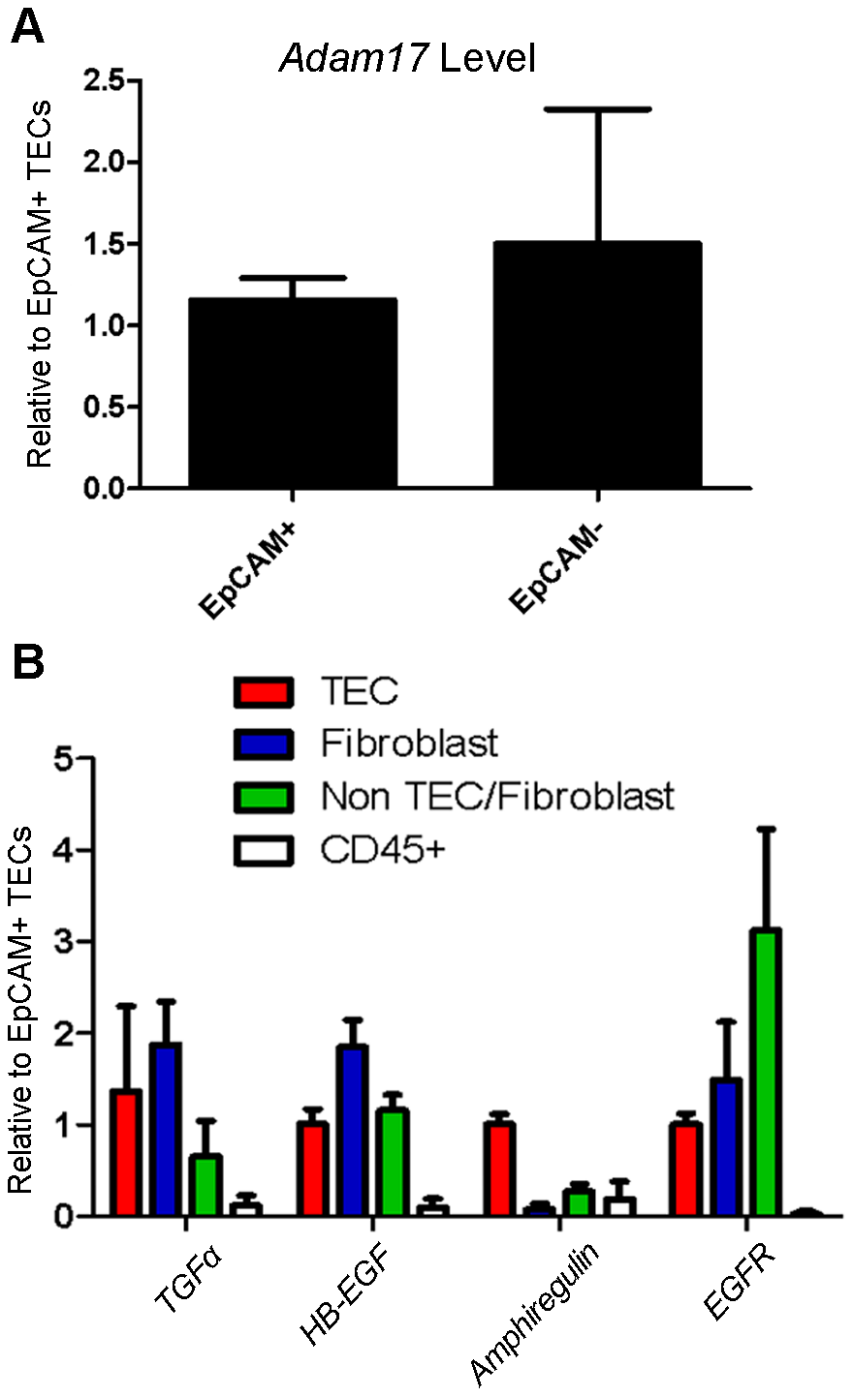
ADAM17 and EGF pathway members are expressed on non-TEC thymic stromal cells. Enzymatically digested thymi from B6 mice were FACS-sorted based on TEC and fibroblast markers. (A) *Adam17* expression on CD45^-^Ter119^-^EpCAM^+^ TEC and CD45^-^Ter119^-^EpCAM^-^ non-TEC stroma with *Gapdh* serving as the internal control. (B) EGF pathway gene expression levels on the indicated populations were calculated relative to levels on TECs with *Gapdh* serving as the internal control. Data are from 2 independent experiments with each experiment consisting of n≥3 pooled mice. Data report mean + SD. In these experiments, the cell types were identified as follows: TEC: CD45^-^Ter119^-^EpCAM^+^MTS15^-^; Fibroblast: CD45^-^Ter119^-^EpCAM^-^MTS15^+^; Non TEC/Fibroblast: CD45^-^Ter119^-^EpCAM^-^MTS15^-^. TECs were sorted to an average of 92% purity, fibroblasts to 68.73% purity, and non-TEC/fibroblast to 95.84% purity. Despite lower fibroblast purity, notably, no sorted fibroblasts were detected within the TEC gate upon re-analysis (data not shown).

Since *Adam17/Foxn1* thymi do not display the T cell developmental block observed in *Adam17* conventional knockout mice [25], it is possible that ADAM17 in another thymic stromal cell type is used during T cell development. To assess this possibility, we examined the non-TEC stroma for presence of ADAM17 and EGF family ligands, which have been shown to be key substrates for ADAM17 *in vivo*. First, to confirm ADAM17 expression on non-TEC stroma, we FACS sorted CD45^−^Ter119^−^EpCAM^−^ cells from normal B6 mice. Quantitative PCR revealed levels of *Adam17* expression in these non-TECs, at least as high as that of TECs (Figure 6A). We then proceeded to examine more specific stromal cell populations, including CD45^−^Ter119^−^EpCAM^−^MTS15^+^ thymic fibroblasts and the CD45^−^Ter119^−^EpCAM^−^MTS15^−^ populations containing thymic endothelial cells and other mesenchyme [35] (Figure 5A). Notably, in thymic fibroblasts, we observed levels of *TGFα* and *HB-EGF* that were elevated relative to those of TECs, as well as the presence of the *EGF receptor* (Figure 6B). This indicates that ADAM17 and EGF pathway substrates shown to be critical in other developmental systems are present on the non-TEC populations in the thymus.

### Adam10 expression is not altered on Adam17/Foxn1 thymic stroma

ADAM10 has a high degree of substrate overlap with ADAM17 and may function to compensate for ADAM17 deficiency *in vivo*. ADAM10 has been shown to be tightly regulated at the transcriptional level, whereas ADAM17 is predominantly activated in a post-transcriptional manner [6], [19]. To assess whether *Adam10* expression is altered in the thymus following deletion of *Adam17,* we performed qRT-PCR on sorted EpCAM^+^ TECs and EpCAM^−^ stroma. We did not see any alteration in *Adam10* levels in TECs or non-TEC stroma in *Adam17/Foxn1* mice (Figure S2).

## Discussion

Loss of *Adam17* results in a profound non-cell autonomous T cell developmental block in conventional knockout mice, and the underlying assumption from the previous literature was that absence of ADAM17 in thymic epithelial cells was the key cause of this block [25]. In the present study, we have conditionally knocked out *Adam17* in thymic epithelial cells expressing the transcription factor *Foxn1* [27]. We have convincingly shown that wildtype *Adam17* is eliminated at the mRNA level by both quantitative and standard RT-PCR strategies. Surprisingly, *Adam17/Foxn1* conditional KO mice do not possess a similar block in T cell development to *Adam17* conventional knockout mice. No hematopoietic lineage was statistically different in percentage or total cell numbers in the *Adam17/Foxn1* thymus compared to controls. We have also performed a limited analysis of embryonic day 17.5 thymi in conventional *Adam17* knockout mice, and have not observed any differences in thymocyte subset or TEC subset frequencies at this earlier developmental stage (Gravano et al., unpublished observations). Therefore, we conclude that TEC subsets are unaltered at either the fetal or adult stages in the absence of *Adam17*. In addition, our results show that unlike the thymus in the postnatal conventional *Adam17* knockout mice [25], the thymus in TEC-specific, *Adam17* knockout mice is not defective in the generation of T cells. Our data support the idea that *Adam17* is a regulator of adult, but not fetal, thymic development, but is not dependent on *Aire* levels. Our results, which show that *Adam17* in TECs is not necessary for thymic development, were contrary to expectation, but could be explained by several possible reasons, described below.

**Figure 7:**
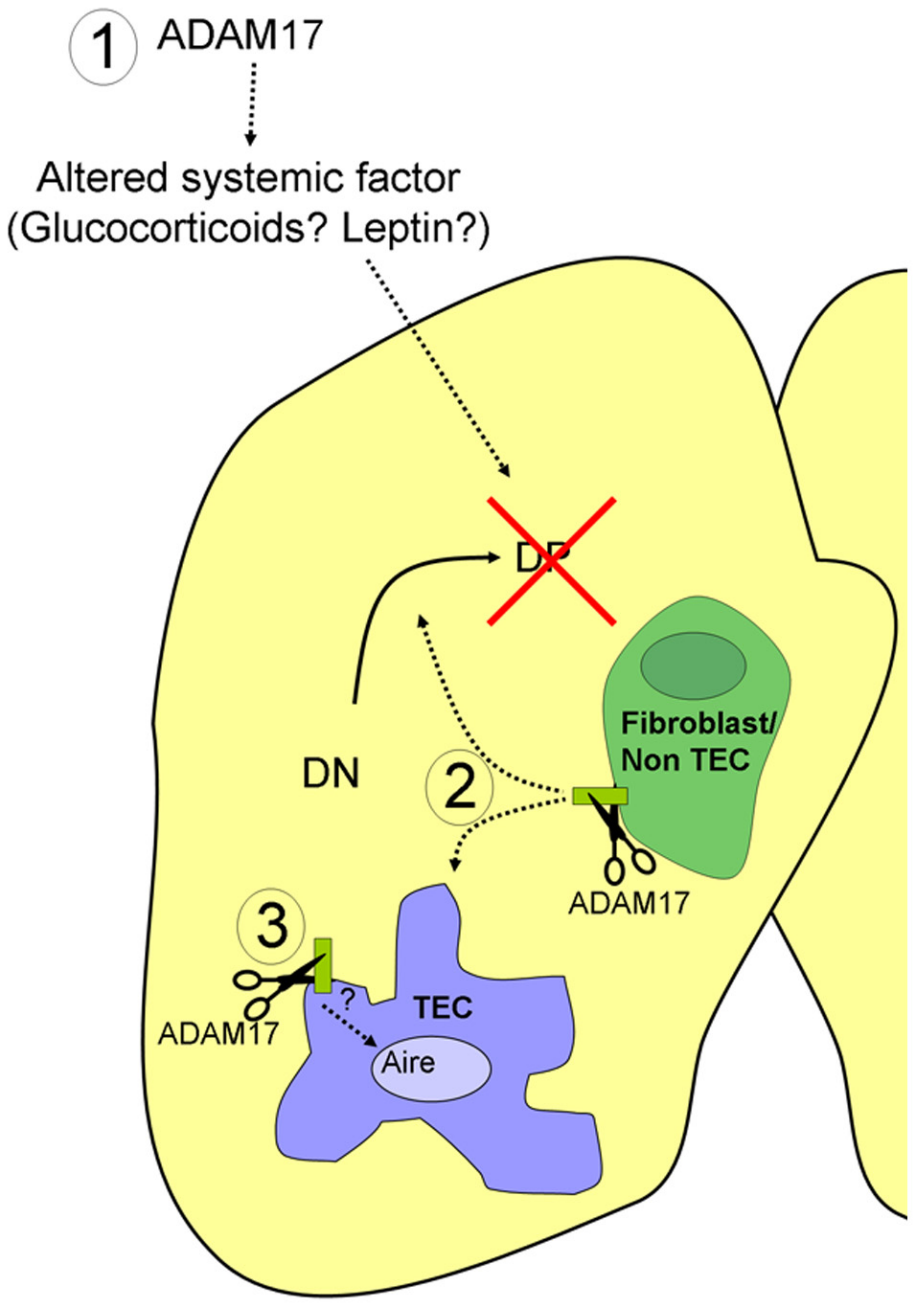
A revised model of ADAM17's role in the thymus. ADAM17 may act in several related manners which collectively regulate T cell development: 1) ADAM17 may modulate systemic factors in that can affect T cell development in an endocrine fashion, potentially through control of DP cell survival; 2) ADAM17 may release factors from non-TEC stromal cells (such as fibroblasts) that in turn act directly on thymocytes or on TECs to promote proper T cell development; 3) ADAM17 in TECs regulates expression of *Aire* mRNA levels through an unknown substrate interaction.

First, ADAM17 may be acting within the thymic microenvironment to regulate T cell development, but on a non-epithelial cell type (see conceptual model shown in Figure 7). Consistent with this idea, we have observed high levels of *Adam17* expression on the CD45^−^EpCAM^−^ population of cells in the thymus, which contain fibroblasts, endothelial cells, and other mesenchymal cells. Furthermore, these non-epithelial cell types are unaffected in *Adam17/Foxn1* mice. In addition, TGFα and HB-EGF, two EGF pathway ligands shed by ADAM17 during the development of other epithelial tissues, are expressed at high levels in adult thymic fibroblasts relative to TECs. It is has been well established that growth factors such as FGF7 and FGF10 are secreted from the neural crest-derived mesenchyme that surrounds the thymic epithelium in fetal development [36]. The interaction of mesenchyme regulates TEC development, proliferation and ability to support thymopoiesis. Through fetal thymic organ culture experiments it has been shown that this mesenchymal support can be replaced by addition of the soluble ADAM17 substrate TGFα [37]. Thus, ADAM17 may potentially be produced by non-TEC stroma during the fetal and/or adult stages to promote the development and proliferation of TECs, facilitating proper thymocyte development. Alternatively, signals shed by ADAM17 on non-TEC stroma may target the thymocytes directly. It has previously been shown that thymic mesenchyme and fibroblasts can play a direct role in early T cell development [38], [39]. Recently, intrathymic PDGFR^+^ pericytes of neural crest origin have been show to promote thymocyte egress, highlighting the importance of non-TEC stroma in thymus physiology [40], [41].

Second, it is possible that there is an extrathymic requirement for ADAM17 for proper T cell development (Figure 7). Since ADAM17 sheds factors that can act systemically [17], it is possible that extrathymically derived factors influence intrathymic development. The multiple systemic defects in *Adam17* ^ΔZn/ΔZn^ mice also support this hypothesis [17]. For example, the DP thymocyte subset, which is the population reduced in *Adam17* ^ΔZn/ΔZn^ mice [25], is highly sensitive to levels of systemic factors such as glucocorticoids, which are involved in metabolism and stress response [42]. Adult *Adam17* ^ΔZn/ΔZn^ mice have a lean and hypermetabolic phenotype characterized by reduced fat mass despite normal food intake. However, corticosterone (the major glucocorticoid in mice) was not altered in *Adam17* ^ΔZn/ΔZn^ mice [24]. Interestingly, plasma levels of leptin, a key factor in the energy homeostasis system, were reduced approximately 2-fold in *Adam17* ^ΔZn/ΔZn^ mice. Leptin-deficient *ob/ob* mice possess a high level of DP thymocyte apoptosis, which can be reversed by addition of recombinant leptin [43]. Leptin has been shown to act by protecting thymocytes from glucocorticoid induced apoptosis [44]. The phenotype of *ob/ob* DP thymocytes is very similar to *Adam17* ^ΔZn/ΔZn^ mice. It remains to be determined if changes in these systemic factors (which would not be deleted in the *Adam17/Foxn1* mice) are the cause of the thymic hypocellularity in *Adam17* ^ΔZn/ΔZn^ mice.

Our data show that *Adam17/Foxn1* mice have reduced expression of the transcription factor Aire in TECs, but this reduction does not affect TEC population composition. Mutations in Aire have been associated with autoimmune disease in humans and in mouse models [29]. *Adam17/Foxn1* mice do not display any overt phenotypes that would indicate autoimmune disease, which is consistent with the normal thymic phenotype and production of mature T cells observed in two-to-three-month-old *Aire* KO mice [45]. The extent of autoimmune disease in *Aire* KO mice is dependent on the genetic background [46], with very mild disease observed on the mixed 129xB6 background [47] (which incidentally, is the same background as the *Adam17/Foxn1* mice used in our studies). Therefore, the unaltered thymus development observed in *Adam17/Foxn1* mice, despite the reduced Aire mRNA expression in these mice, is of similar phenotype to *Aire* KO mice. Little is known about the factors that regulate Aire expression. Aire expression is highly specific to mature medullary TECs only at their latest stages of differentiation [33]. Lymphotoxin β receptor signaling was initially thought to regulate the expression of Aire [48], but the current consensus now favors the conclusion that lymphotoxin β receptor signals are required for TEC differentiation but do not impact Aire expression directly [29], [49]. We are convinced that our PCR primers should detect all known *Aire* splice variants [50], strongly supporting the idea that *Adam17* promotes the transcription of the *Aire* gene. Aire protein expression was measured both by flow cytometry and immunohistochemistry, using different antibodies but with similar results, demonstrating that the Aire protein levels are close to normal in *Adam17/Foxn1* mice. Therefore, one explanation is that low level of *Aire* mRNA transcript is sufficient to produce normal Aire protein levels in *Adam17/Foxn1* mice. Alternatively, *Adam17* may also play a role in the turnover of Aire protein. If ADAM17 degrades Aire protein as part of its normal function, then we might indeed expect to see the levels of Aire protein to be normal in *Adam17/Foxn1* mice even if *Aire* mRNA levels are low, due to an increased lifespan of the Aire protein in the absence of *Adam17*. The role of ADAM17 as sheddase and an activator of regulated intramembrane proteolysis is well documented [5], [7], [8], [9], but to date, the substrates of ADAM17 have been restricted to extracellular transmembrane receptor and ligands. In contrast, no direct link between ADAM17 and the regulated turnover of intracellular nuclear proteins, such as Aire, has been reported. Therefore, further experimentation is required to confirm whether or not ADAM17 can facilitate Aire protein turnover in mTECs.

ADAM17 has recently been shown to cleave the RANK receptor in macrophages [51]. RANK signaling is a key signaling pathway in the development of mature medullary Aire-expressing TECs at the fetal stage [52], [53], [54]. Although RANKL is necessary for the induction of Aire expression and mTEC development in fetal thymic organ cultures, the dependence of RANKL on mTEC after the embryonic stage is not as severe [52]. Our data show that adult mTEC frequencies are not affected, and Aire^+^ TECs appear to be mature in the absence of *Adam17*. Furthermore, although *Aire* mRNA levels are severely reduced in adult *Adam17/Foxn1* mice, Aire protein levels are not (Figure 4). Therefore, despite the link between ADAM17 and RANK signaling, our observation that TEC populations are normal in *Adam17/Foxn1* mice does not support the hypothesis that ADAM17 is necessary to facilitate RANK signaling in the adult thymus.

In conclusion, our findings reveal that ADAM17 is dispensable in TECs for the ability to support T cells to progress through the characterized stages of αβ T cell development. This contrasts with conventional *Adam17* ^ΔZn/ΔZn^ mice and suggests a novel role for ADAM17 in non-TEC stroma or extrathymically-derived ADAM17 substrates on T cell development. *Adam17* has been deleted under the control of other promoters including *Sox9*, resulting in an osteoporotic phenotype due to G-CSF dysregulation [55] and *Tie2,* revealing a role for *Adam17* in pathological neovascularization [56]. New conditional knockout strategies are necessary to identify the cell type in which ADAM17 non-autonomously regulates T cell development. In addition, our findings indicate that ADAM17 in TECs regulates the expression of the transcription factor *Aire*. Further experiments will be required to identify the exact ADAM17 substrates involved in *Aire* regulation and whether and the ADAM17 deficit in TECs leads to breaches in the regulation of negative selection in thymocytes and self-tolerance.

## Materials and Methods

### Ethics Statement

All animal procedures were approved by the UC Merced Institutional Animal Care and Use Committee (Protocol # UCM 185).

### Mice

*Adam17^flox/flox^* mice [23] and *Foxn1-Cre* mice [27] have been previously described. *Adam17^flox/flox^* mice were crossed with *Foxn1-Cre* mice to generate *Foxn1-Cre/Adam17^flox/+^* mice, which were backcrossed with *Adam17^flox/flox^* mice to generate *Foxn1-Cre/Adam17^flox/flox^* conditional knockouts (referred to as *Adam17/Foxn1)*. *Adam17/Foxn1* mice and littermate controls were of a mixed 129Sv and C57BL/6 (B6) background. Genotyping for Cre recombinase was performed using the following primers: forward 5′-CGATGCAACGAGTGATGAGG-3′ and reverse 5′-GCATTGCTGTCACTTGGTC-3′. Cre-expressing mice were distinguished by the presence of a 288bp amplicon from ear punch DNA samples. Genotyping for *Adam17* was performed using the following primers: forward 5′-GCCACGATTCAGAGTGATTCC-3′ and reverse 5′-CTCCATCAGCAGTTAAGAGCAC-3′ which allowed clear distinction between the *Adam17^+^* and *Adam17^flox^* alleles. B6 mice were purchased from Jackson Laboratories. In our experiments, control mice were of either the genotype *Adam17^flox/+^* or *Adam17^flox/flox^* (without Foxn1-Cre). We have confirmed that no differences in thymocyte and TEC lineages exist between *Foxn-1-Cre/Adam17^+/+^* and control mice (data not shown). Mice were housed pathogen free in sterile microisolator cages under 12 hour light/dark cycles with ad libitum access to sterile feed and autoclaved water. Mice were euthanized by CO_2_ asphyxiation followed by cervical dislocation.

### Thymocyte and TEC isolation

Thymi were harvested and placed in Medium 199 (M199) (Invitrogen) containing 2% heat inactivated fetal calf serum (FCS) (Atlanta Biologicals). For thymocyte analysis, thymocytes were extracted by mechanical disruption with the base of a 5 ml syringe followed by filtration through 70 µm nylon mesh. For TEC analysis by flow cytometry [57], thymi were trimmed of excess fat and connective tissue. Thymi were placed in M199 and the capsule was nicked in several places with fine scissors. Thymi were agitated with a magnetic stirrer at 4°C for 20 minutes to remove a large fraction of thymocytes. Thymic fragments were then transferred into an enzymatic digestion buffer containing 0.125% (w/v) Collagenase D (Roche) with 0.1% (v/v) DNAse I (Roche) and gently rotated on an orbital shaker at 37°C for 15 minutes. Thymic fragments were pelleted by centrifugation at 300× g and the digestion buffer was exchanged. After 4 digestions, 0.125% (w/v) dispase (Worthington Biochemical) was added to the digestion buffer and thymi were incubated for an additional 15 minutes. After the final digestion, cells were incubated in PBS containing 5 mM EDTA + 1% FCS + 0.02% (w/v) NaN_3_ for 10 minutes on ice. Cells were passed several times through a 21G needle and filtered through a 70 µm nylon mesh. Red blood cells were lysed with ACK lysis buffer (0.15 M NH_4_Cl + 10 mM KHCO_3_ + 0.1 mM Na_2_EDTA, pH 7.2). Cells were counted by hemocytometer and population numbers determined by calculating based on flow cytometric gating.

### Antibodies, Flow Cytometry, and Cell Sorting

The anti-EpCAM (clone G8.8, Developmental Studies Hybridoma Bank, University of Iowa) hybridoma was cultured in DMEM media containing 10% FCS until confluent. Hybridoma supernatants were collected and antibodies purified using goat anti-rat CNBr Sepharose Beads (Jackson Immunohistochemical, Amersham Biosciences). EpCAM (rat IgG2a) antibody was conjugated to phycoerythrin using the Lightning Link PE kit (Novus Biological). The following primary antibodies were purchased from Biolegend: anti-MHCII-PE/Cy5 (clone MM5/114.15.2), anti-CD80-FITC (clone 16-10A1), anti-CD45-APC/Cy7 (clone 104), anti-CD4-PE/Cy7 (clone RM4-5), anti-CD8α-FITC (clone 53–6.7), anti-CD44-PE (clone IM7), anti-CD25-APC (clone PC61), anti-TCRγδ-FITC (clone UC7-13D5), anti-TCRβ-PE (clone H57-597), anti-CD3-APC (clone 145–2C11), anti-CD19-PE/Cy7 (clone 6D5), anti-Ter119 (clone Ter-119). The anti-FoxP3-PE antibody (clone FJK-16s) was purchased from eBioscience. Purified MTS-15 antibody was a gift of Dr. Richard Boyd, Monash University, Clayton, Victoria, Australia. Purified anti-Aire antibody (clone 5H12-2 ) was a gift of Dr. Daniel Gray, Walter and Eliza Hall Institute, Melbourne, Australia. Rat IgG2c isotype control (clone RTK 4174) was purchased from Biolegend. The lectin Ulex agglutitin-1 (UEA1)-FITC was purchased from Vector Laboratories. The secondary antibody anti-rat IgG2c-biotin (clone MRG2c-67, Biolegend) was used to label MTS-15 or anti-Aire, followed by staining with Streptavidin-FITC (eBioscience) or Streptavidin-PE/Cy5 (Biolegend). For TEC analysis and sorting, CD45^+^ cells were first depleted by magnetic activated cell sorting (MACS). Cells were stained with anti-CD45 coated microbeads (Miltenyi Biotech) and magnetically sorted using the “DepleteS” program on an AutoMACS cell sorter (Miltenyi Biotech). Prior to antigen staining, Fc receptors were blocked with anti-CD16/32 (eBioscience). Each stain was performed on ice for 15 minutes in FACS staining buffer containing 1x HBSS, 4.2 mM sodium bicarbonate, 0.1% w/v sodium azide and 1% bovine serum albumin, Fraction V, pH 7.2. For thymic stromal analysis, the MTS-15 stain was performed first, then washed and stained with fluorochrome-conjugated primary antibodies and streptavidin-FITC or PE/Cy5. Labeled cells were resuspended in FACS staining buffer containing 0.1 µg/ml DAPI to assess viability of unfixed cells. For Aire and FoxP3 intracellular stain, surface staining was performed first, followed by fixation and permeabilization utilizing the buffers in the Foxp3 Staining Set (eBioscience) according to the manufacturer's instructions. For TEC isolation, flow cytometric analysis and sorting of the CD45^-^ fraction was performed on a FACSAria cytometer (BD Biosciences). Thymic populations were sorted to greater than 95% purity, unless noted otherwise. Flow cytometric data analysis was performed using FlowJo software (TreeStar).

### RT-PCR and qRT-PCR

Total RNA was extracted from FACS or MACS sorted cells using the RNeasy kit (Qiagen) and cDNA was generated using the Superscript III RT kit (Invitrogen) according to the manufacturers' instructions. Reverse transcriptase PCR (RT-PCR) primers to *Adam17* were designed to span the floxed exon2 with the forward primer 5′-CGGAGGAAGCAGGCTCTG-3′ annealing to exon1 and the reverse primer 5′-GAGTCAGGCTCACCAACCAC-3′ annealing to exon3. This allows discrimination of a wildtype transcript and a mutant, null transcript resulting from recombination. After one minute dissociation at 95°C, *Adam17* was amplified from the cDNA over 40 cycles at 95°C for 30 seconds, 57°C for 30 seconds, and 72°C for 1 minute. Quantitative RT-PCR (qRT-PCR) was performed using SYBR Green PCR master mix (Applied Biosystems) and samples run on an Applied Biosystems 7300 Real-Time system. Samples were denatured at 95°C for 2 minutes followed by 40 cycles of 95°C for 15 seconds and 60°C for 1 minute. The ΔΔCt method was utilized to determine relative expression levels of the gene of interest between samples using ribosomal protein L7 (RPL7) as the internal control. For *Adam17* qRT-PCR the above noted forward primer was used in conjunction with the reverse primer 5′-GTTTCTAAGTGTGTCGCAGACTG-3′ that anneals to exon2. Therefore, amplification only occurs off the wildtype transcript. The following other primer sequences were used (forward then reverse): *Rpl7*, 5′-GAAGCTCATCTATGAGAAGGC-3′ and 5′-AAGACGAAGGAGCTGCAGAAC-3′ [58]; *Adam10*, 5′- CACTGCCGGGGAGGCTCGTCG-3′ and 5′- CCGCCTCCTCACGGGTTAACCG-3′; *Aire*, 5′-GTACAGCCGCCTGCATAGC-3′ and 5′-CCCTTTCCGGGACTGGTTTA-3′; *Caseinα*, 5′-CCTATGAGTGTAGTGGATCAGGCA-3′ and 5′-AGGCATCATACTGGAAGATTTGTG-3′; *Caseinβ*, 5′-TGTGCTCCAGGCTAAAGTTCACT-3′ and 5′-GGTTTGAGCCTGAGCATATGG-3′; *Caseinγ*, 5′-ATGTTGCACACCTCTTCACCAG-3′ and 5′-GGCGTGTTATGGATGGCATT-3′; *Caseinκ*, 5′-GCCGTGGTGAGAAGAATGACA-3′ and 5′-AAGTTCAGGACGGAGCGGA-3′; *Insulin2*, 5′-GGGAGTCCCACCCCACCCAG-3′ and 5′-CACGCTCCCCACACACCAGG-3′; *Gad67*, 5′-GGTTCGCACAGGTCACCC-3′ and 5′-GCCATTCACCAGCTAAACCAA-3′; *Spt1*, 5′-CTGGTGAAAATACTGGCTCTGAA-3′ and 5′-AGCAGTGTTGGTATCATCAGTG-3′. Unless noted otherwise, primer sequences were obtained from Primerbank (http://pga.mgh.harvard.edu/primerbank/) or from previous literature [30]. All qRT-PCR primers were validated to be between 90–110% efficient by a template serial dilution standard curve and to have a single peak when a dissociation curve was performed.

### Immunohistochemistry

Thymi were extracted and snap frozen in OCT compound (Sakura Finetek) over dry ice. Sectioning of the thymus, tissue processing protocols, keratin-5, keratin-8 and Aire staining and morphometry data analysis were performed as described [59].

### Statistical Analysis

Differences between means were tested using two-tailed T-test (GraphPad Prism) and were considered significant if p<0.05 (*), p<0.01 (**), p<0.001 (***).

## Supporting Information

Figure S1 *Thymic and splenic regulatory T cells are present in Adam17/Foxn1 mice.* Thymus and spleen from 11-12 week old mice were harvested. FoxP3 intracellular stain was performed and the FoxP3+ fraction was determined within the CD4SP thymic population (A, left and middle panels). CD25 stain was performed to assess co-localization with FoxP3 stain on CD4SP thymocytes (A, right panels). Splenic CD4SP lymphocytes assessed for FoxP3 expression (B). Frequency of FoxP3+ thymocytes within the CD4SP gate (C). All flow plots are representative of indicated groups. Data represent mean + SD; CD4SP, CD4 single positive; Control, n = 5; Adam17/Foxn1, n = 3. *Control: fl/+ or fl/fl; Adam17/Foxn1: Cre fl/fl*.
(3.66 MB TIF)

Figure S2 *Adam10 levels are unaltered on ADAM17/Foxn1 TECs and non-TEC stroma.* FACS-sorted CD45-Ter119-EpCAM+ TECs and CD45-Ter119-EpCAM- stroma were pooled from 8-week old *Control* and *Adam17/Foxn1* mice (n≥3 for each pool). *Adam10* levels were determined in TECs (A) and non-TEC stroma (B). *Rpl7* was used as the internal control and expression levels were determined relative to mean expression in *Control* mice. Data represent mean + SD of 2 independent experiments. No statistically significant differences were found. *Control: fl/+ or fl/fl; Adam17/Foxn1: Cre fl/fl*.
(0.08 MB TIF)
