# Supplementary figures and images for: ADAM17 Deletion in Thymic Epithelial Cells Alters Aire Expression without Affecting T Cell Developmental Progression
Source: PLoS One. 2010 Oct 20;5(10):e13528. doi: 10.1371/journal.pone.0013528 (PMC2958126; doi:10.1371/journal.pone.0013528)

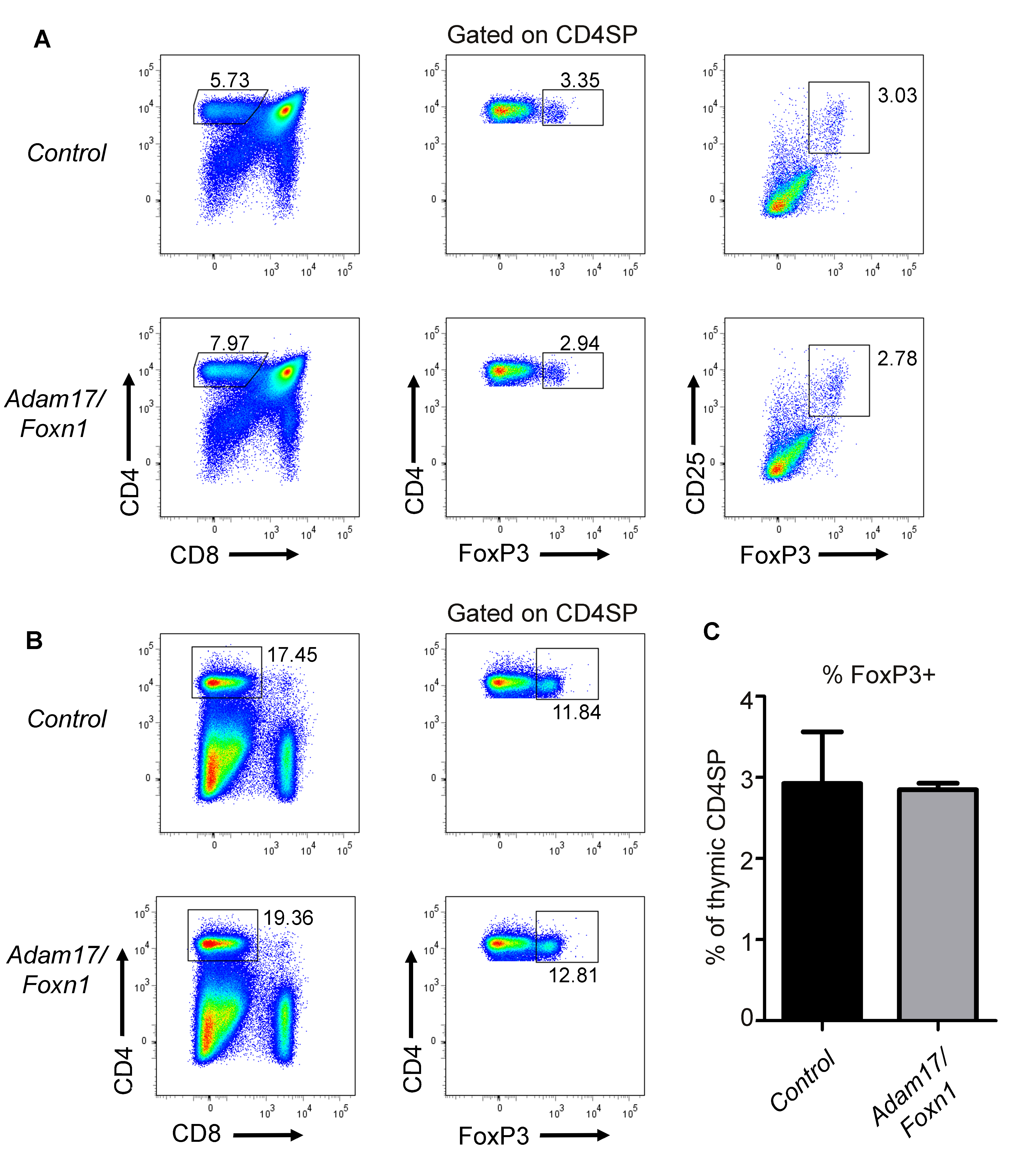

Supplement: Figure S1 — Thymic and splenic regulatory T cells are present in Adam17/Foxn1 mice. Thymus and spleen from 11-12 week old mice were harvested. FoxP3 intracellular stain was performed and the FoxP3+ fraction was determined within the CD4SP thymic population (A, left and middle panels). CD25 stain was performed to assess co-localization with FoxP3 stain on CD4SP thymocytes (A, right panels). Splenic CD4SP lymphocytes assessed for FoxP3 expression (B). Frequency of FoxP3+ thymocytes within the CD4SP gate (C). All flow plots are representative of indicated groups. Data represent mean + SD; CD4SP, CD4 single positive; Control, n = 5; Adam17/Foxn1, n = 3. Control: fl/+ or fl/fl; Adam17/Foxn1: Cre fl/fl. (3.66 MB TIF) [file pone.0013528.s001.tif]

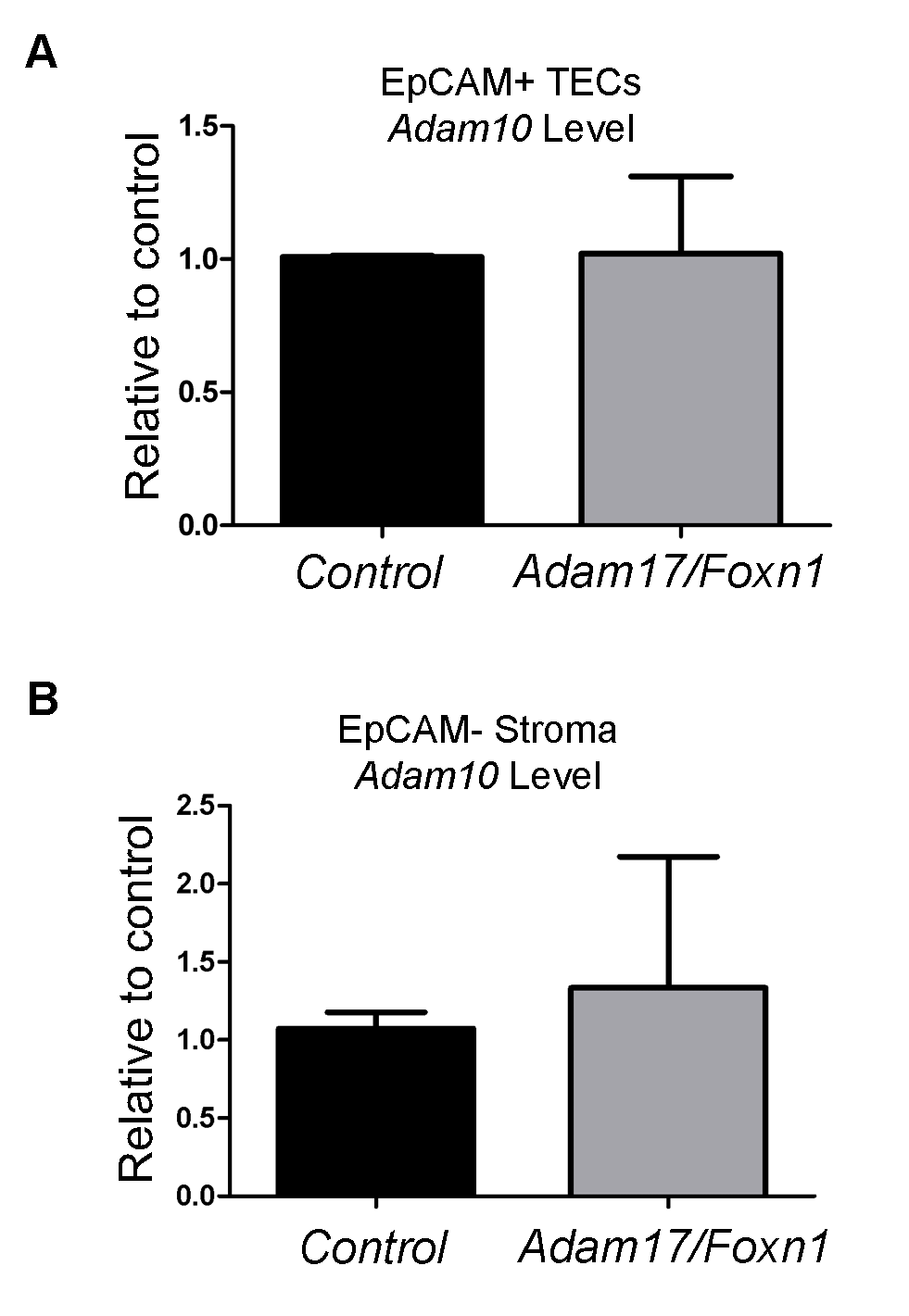

Supplement: Figure S2 — Adam10 levels are unaltered on ADAM17/Foxn1 TECs and non-TEC stroma. FACS-sorted CD45-Ter119-EpCAM+ TECs and CD45-Ter119-EpCAM- stroma were pooled from 8-week old Control and Adam17/Foxn1 mice (n≥3 for each pool). Adam10 levels were determined in TECs (A) and non-TEC stroma (B). Rpl7 was used as the internal control and expression levels were determined relative to mean expression in Control mice. Data represent mean + SD of 2 independent experiments. No statistically significant differences were found. Control: fl/+ or fl/fl; Adam17/Foxn1: Cre fl/fl. (0.08 MB TIF) [file pone.0013528.s002.tif]
